# Is a low Functional Movement Screen score (≤14/21) associated with injuries in sport? A systematic review and meta-analysis

**DOI:** 10.1136/bmjsem-2018-000501

**Published:** 2019-09-18

**Authors:** Manuel Trinidad-Fernandez, Manuel Gonzalez-Sanchez, Antonio I Cuesta-Vargas

**Affiliations:** 1 Physiotherapy, Universidad de Málaga, Málaga, Spain; 2 Physiotherapy, Human Physiology and Anatomy, Vrije Universiteit Brussel, Brussels, Belgium; 3 Institute of Health & Biomedical Innovation, Queensland University Technology, Brisbane, Queensland, Australia

**Keywords:** Functional movement screen, injury, risk, prediction

## Abstract

**Objective:**

To assess whether Functional Movement Screen (FMS) score is associated with subsequent injuries in healthy sportspeople.

**Design:**

Systematic review and meta-analysis.

**Data sources:**

The following electronic databases were searched to December 2017: Medline, PubMed, PsycINFO, SPORTDiscus, Cumulative Index of Nursing and Allied Health Literature, Scopus, Embase, and Physiotherapy Evidence Database.

**Eligibility criteria for selecting studies:**

Eligibility criteria included (1) prospective cohort studies that examined the association between FMS score (≤14/21) and subsequent injuries, (2) a sample of healthy and active participants without restrictions in gender or age, and (3) the OR was the effect size and the main outcome.

**Results:**

Thirteen studies met the criteria for the systematic review and 12 were included in the meta-analysis. In 5 of the 12 studies, and among female athletes in 1 study, FMS score ≤14 out of 21 points was associated with subsequent injuries. The overall OR of the selected studies in the meta-analysis was 1.86 (95% CI 1.32 to 2.61) and showed substantial heterogeneity (I^2^=70%).

**Summary/Conclusion:**

Whether or not a low FMS score ≤14 out of 21 points is associated with increased risk of injury is unclear. The heterogeneity of the study populations (type of athletes, age and sport exposure) and the definition of injury used in the studies make it difficult to synthesise the evidence and draw definitive conclusions.

**Trial registration number:**

CRD42015015579.

**What is already known?**

- The Functional Movement Screen (FMS) is a popular screening tool, and there are claims that in certain sports lower scores can identify players at greater risk of injury.

**What are the new findings?**

- In half of the published studies, a baseline FMS score of ≤14 out of 21 points was associated with a greater risk (OR) of injury.
- The heterogeneity of the study populations (type of athletes, age and sport exposure) and the definition of injury used in the studies make it difficult to synthesise the evidence and draw definitive conclusions.

## INTRODUCTION

Sports activity is associated with an injury.^1 2^ Sports injuries have an incidence rate of 26–34 injuries per 1000 persons in the USA and the European Union.^3 4^ An assessment test is usually performed at the beginning of each season as a strategy to manage the risk of injury.^5 6^ The early detection of injuries and the development of support programmes could be useful in preventing injuries and the disruption of sports practice.^7^ Therefore, it is an important aim of clinicians, sports professionals and researchers.^8 9^ Several screening tools for injuries to the ACL of the knee, hamstring, groin and ankle could be recommended for use in the field.^10^ One of these screening tools for injuries is the Functional Movement Screen (FMS).^11^

The FMS is an assessment tool which identifies the quality of movement and requires both balance and stability.^11^ It is popular in many fitness and rehabilitation settings.^12^ Seven basic exercises are scored from 0 to 3 according to the grading criteria: deep squat, inline lunge, hurdle step, shoulder mobility, active straight leg raise, trunk stability push-up and rotary stability.^11^ The FMS test could identify movement ability, and then suggest exercises based on the dysfunctions and limitations detected^13 14^ which positively influence strength and flexibility.^15^ The FMS has good intrarater (intraclass correlation coefficient (ICC)=0.74–0.8) and inter-rater (ICC=0.9–0.97) reliability.^16^ The relevance of this test is increasing due to its proposed injury-predictive ability in different sports^17 18^ and at different ages.^19 20^

Kiesel *et al* 9 were the first to note that subjects who had a total score of less than 14 out of 21 points were more likely to suffer an injury during a sports season. From this study to date, some authors have investigated in prospective studies if the FMS is associated with injuries using this cut-off.^21 22^ Meanwhile, other researchers performed a sensitivity and specificity analysis and then chose the cut-off for their study.^23^ After an analysis of all the studies in the systemic review, 14 out of 24 chose a cut-off of 14/21; hence, the use of this cut-off may still be questionable.^23^

In the last few years, four systematic reviews have been published with the intention of analysing the association between suffering from lesions and the FMS. Specifically, Moran *et al* 23 and Bunn *et al* 24 published systematic reviews that included 26 and 20 articles, respectively. The first study applied a cut-off range of between 11 and 17 out of 24 points, including one study with several cut-offs and four studies where the cut-off was not indicated. The second study included a range of cut-offs from 14 to 17 out of 24 points. Bonazza *et al* 25 published a study where the FMS was analysed and included a quantitative (nine studies) and qualitative (five studies) analysis. Dorrel *et al* 26 published another systematic review; however, they did not analyse the cut-offs with special attention and did not measure the homogeneity of the meta-analysis. None of the studies consulted has explicitly and uniquely used the original cut-off that had been previously suggested (14/21) to analyse the ability of the FMS to identify a high risk of suffering from injury in healthy people. For this reason, the aim of this systematic review is to assess the association between the FMS score and subsequent injuries in healthy people by applying a cut-off of 14/21. Furthermore, it aims to perform a meta-analysis of the data from the selected studies.

## METHODS

### Design

This systematic review and meta-analysis is reported according to the Preferred Reporting Items for Systematic Reviews and Meta-Analyses statement.^27^

### Search strategy

The electronic databases Medline, PubMed, PsycINFO, SPORTDiscus, Cumulative Index of Nursing and Allied Health Literature, Scopus, Embase, and Physiotherapy Evidence Database were searched and the last search was conducted on 1 June 2019. No hand searches were conducted. The search was limited to studies published in the last 10 years (2007–2019) because the seminal study by Kiesel *et al* 9 was published in 2007. The keywords searched in the titles and abstracts were ‘functional movement screen’, ‘risk’, ‘injury’ and ‘predict’. The following search strategy was developed by the authors: functional movement screen* AND (predict OR prediction OR risk OR injury).

### Study criteria

MT-F and AIC-V reviewed the screening phase of the studies independently to confirm whether the inclusion and exclusion criteria were met. Disagreements were resolved by consensus. The inclusion criteria were (1) prospective cohort studies that examined the association of injury and FMS score, and (2) samples of healthy and active subjects without restrictions in gender and age. The OR was chosen as the effect size and the main outcome because it was a quantitative risk of injury between groups.

The first exclusion of potential items found in the databases was made when reading the title and abstract of the articles. The following were the first exclusion criteria: (1) secondary research, (2) studies that were not related to the FMS or injury risk, and (3) studies that were not published in English. Subsequently, the full text of the selected studies was read. The following were the second exclusion criteria: (1) not prospective studies, (2) studies where the cut-off was not 14/21, and (3) studies where it was impossible to know the OR. The quality of the remaining articles was critically evaluated with an assessment tool.

According to the meta-analysis, the selected studies for the systematic review were excluded if there was not enough information to know the number of injuries according to their score.

### Data extraction

The methodological appraisal tool was used on the selected studies by the two researchers (MT-F and AIC-V). The information extracted was author information, year of publication, number of samples and setting of subjects, anthropometric data available, intervention, follow-up, number of injuries, injury definition, diagnosis outcomes (sensitivity, specificity and OR if they were indicated), and conclusion. The main summary measure was the calculated OR. The results obtained using the 14/21 cut-off were extracted. If possible, it was divided between men and women to better analyse the results.

### Risk of bias

The two reviewers (MT-F and AIC-V) critically evaluated the selected studies using the Strengthening the Reporting of Observational Studies in Epidemiology (STROBE) statement for cohort studies, which was used to assess the potential risks of studies; the total score was 22.^28^ STROBE analyses the title, abstract, background, objectives, study design, setting, participants, variables, data sources, bias, study size, statistical methods, participants, descriptive and outcome data, results, limitations, and discussion.^28^ Studies that had half of the total score (≥11) were chosen. When a consensus was not reached, a third reviewer arbitrated.

### Statistical analyses

The OR with 95% CI was extracted from the studies with results of sensitivity, specificity and area under the receiver operating characteristic (ROC) curve. The area under the ROC curve classifies the performance measure when compared with the overall accuracy.^29^ Where the OR according to the 14/21 cut-off was not calculated, it was then calculated using a contingency table with data provided by the author. The table was completed using the stipulated formulas of sensitivity, specificity and the OR calculation by the Meta-DiSc software (Unidad de Bioestadística Clínica del Hospital Ramón y Cajal, Madrid, Spain). Age and anthropometric characteristics were shown with mean and SD. For statistical analysis, SPSS V.17.0 software for Windows was used. The results were calculated by information from the study if they did not show the number of subjects in each group.

For the meta-analysis, the Meta-DiSc software was also used to determine the overall OR of the selected studies. Statistical heterogeneity was assessed with Cochran’s Q test and the forest plot by the same software. Then, I^2^ statistic was calculated to quantify the heterogeneity. The following cut-off parameters for the I^2^ statistics were used: may not represent important heterogeneity, 0%–40%; may represent moderate heterogeneity, 30%–60%; may represent substantial heterogeneity, 50%–90%; and may represent considerable heterogeneity, 75%–100%.^30^ The results of the meta-analysis are acceptable if the heterogeneity level reaches 0%–40%. A 2×2 contingency table was created to show all the subjects of the selected studies for the meta-analysis. The level of significance was p≤0.05.

## RESULTS

### Selection of studies

Ninety-three studies were initially found using the above-mentioned limits and keywords. Twenty duplicate studies in the databases were removed. Forty-two studies were excluded after reading the title and abstract according to the first exclusion criteria. The full text of the remaining 25 studies was read. Twelve studies were removed because they did not meet the second exclusion criteria. Then, the methodological quality of the selected studies was assessed, choosing studies which had a score that was equal to or greater than 11 according to the STROBE statement. Thirteen studies exceeded the appraisal tool and no study was excluded.^9 31–42^ The study scores are found in table 1.

**Table 1:** Assessment of methodological quality of the studies selected: 22 items following the STROBE reporting guidelines

	Title and abstract	Background	Objectives	Study design	Setting	Participant criteria	Variables	Data sources	Bias	Study size	Quantitative variables	Statistical methods	Participants during the study	Descriptive data	Outcome data	Main results	Other analyses	Key results	Limitations	Interpretation	Generalisability	Funding	Total
Mokha *et al* 41	+	+	+	+	+	−	+	+	+	−	+	+	−	+	+	+	+	+	+	+	+	−	18
O’Connor *et al* 35	−	+	+	+	+	−	+	+	+	+	+	+	−	+	+	+	+	+	+	+	−	+	18
Hotta *et al* 39	−	+	−	+	−	+	+	+	+	+	+	+	+	−	+	+	+	+	+	+	+	−	17
Warren *et al* 40	+	+	+	−	−	+	+	+	−	−	+	+	+	+	+	+	+	+	+	+	+	−	17
Bond *et al* 36	−	+	+	+	−	+	+	+	−	−	+	+	−	+	+	−	+	+	+	+	+	+	16
Chorba *et al* 32	−	+	−	−	+	+	+	+	−	−	+	+	+	+	+	+	+	+	+	+	+	−	16
Dossa *et al* 33	+	+	+	+	−	−	+	+	+	−	+	+	−	+	+	+	+	+	+	+	−	−	16
Kodesh *et al* 38	−	+	−	−	+	+	+	+	−	+	+	+	−	+	−	+	+	+	+	+	+	+	16
Knapik *et al* 31	−	+	−	+	+	−	+	+	+	−	+	+	−	+	+	+	+	+	−	+	+	−	15
Butler *et al* 34	−	+	+	−	+	+	+	+	−	−	+	+	−	−	−	+	+	+	+	+	+	−	14
Bushman *et al* 37	+	+	−	−	−	+	+	+	−	−	+	+	−	+	+	+	+	+	−	+	+	−	14
Garrison *et al* 42	+	+	+	−	−	+	+	+	−	−	+	+	−	−	−	+	+	+	+	+	+	−	14
Kiesel *et al* 9	−	+	−	−	−	−	+	+	−	−	+	+	−	−	+	+	+	+	+	+	−	−	11

+, met the criteria; −, did not meet the criteria.

STROBE, Strengthening the Reporting of Observational Studies in Epidemiology.

**Figure 1:**
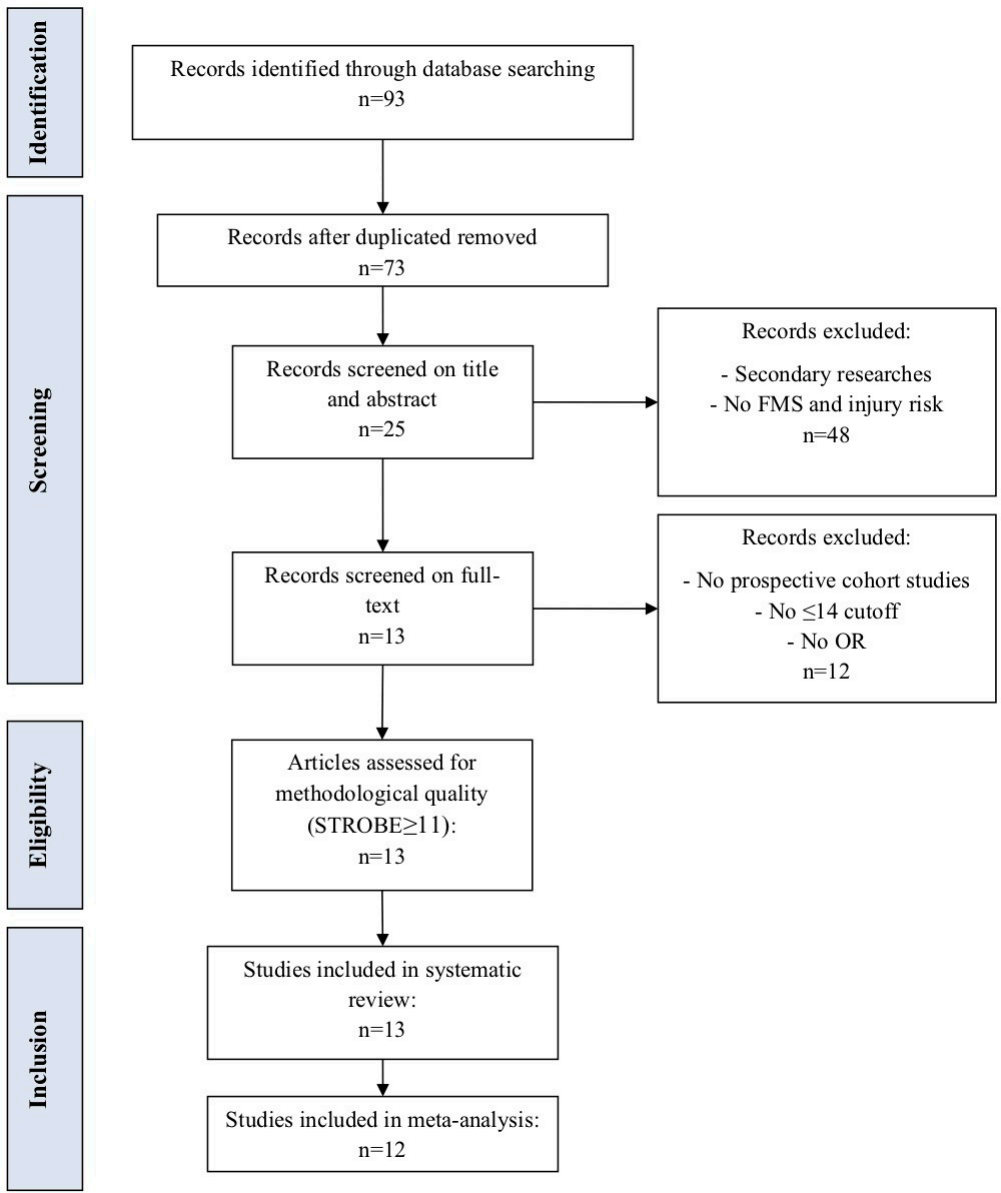
Flow chart through the different phases of study selection. FMS, Functional Movement Screen; STROBE, Strengthening the Reporting of Observational Studies in Epidemiology.

Finally, 12 studies were included in the meta-analysis according to the criteria explained above.^9 31–41^ The study selection process is shown in a flow chart in figure 1.

### Summary of participants and injuries

A comparison of the samples of the selected studies and definition of injury is found in table 2. The total number of participants involved in the selected studies is 5219. There are only 749 female participants. There are subjects from different sports or with good physical condition: soldiers, firemen, coast guards and marine officers. The mean age (±SD) of subjects was between 17 and 22.4 years. Three studies did not show the average age of their sample.

**Table 2:** Study characteristics

	Gender	n	Age (±SD)	BMI (±SD)	Subjects	Injury definition
Bond *et al* 36	Male	63	21.0(±1.4)	24.1	NCAA Division IIcollegiate basketballplayers	Injuries were determined as those that resulted in zero days of time lost, which meant that theplayer returned to full participation on the same day the injury occurred as minimum.
	Female	56	20.2(±1.4)	22.7		
Bushman *et al* 37	Male	2476	–	–	Soldiers	All inpatient and outpatient medical encounters were collected as an injury. Overuse injury related to musculoskeletal conditions, such as stress fractures, Achilles tendinitis or knee pain syndromes. Traumatic injuries such as acute sprains and strains, fractures and dislocations.
Butler *et al* 34	Male	108	–	–	Firefighter trainees	Missing three consecutive days of training in the academy due to musculoskeletal pain, excluding burns.
Chorba *et al* 32	Female	38	19.2 (±1.2)	–	NCAA Division II collegiate athletes	Injury occurred in an organised intercollegiate practice or competition setting. It required medical attention, or the athlete sought advice.
Dossa *et al* 33	Male	20	18.2 (±1.3)*	25.2	Major junior hockey team	Injury occurred during a game or practice which resulted in the player missing at least one game.
Garrison *et al* 42	Male	88	17.0-22.0	-	NCAA Division I collegiate athletes	Injury was defined as any musculoskeletal pain complaint, on or off the field of com- com petition. The iInjury was associated with athletic participation, required consultation with a trainer, physical therapist or physician, and resulted in modified training for at least 24 hours.
	Female	80				
Hotta *et al* 39	Male	84	20.0 (±1.1)	19.7	Runners	Musculoskeletal injury occurred as a result of participating in a practice or race in track and field and was sufficiently severe to prevent participation for at least 4 weeks.
Kiesel *et al* 9	–	46	–	–	Professional football players	Time loss of 3 weeks.
Knapik *et al* 31	Male	770	18.1(±0.7)	23.6(±3.2)	US Coast Guard cadets	Any physical damage to the body that resulted in a clinic visit and that was suspected to have been caused by physical training.
	Female	275	17.9(±0.7)	22.6(±2.7)		
Kodesh *et al* 38	Female	158	19.0	20.8	Soldiers	Diagnosis of an injury was provided by the base medical physician.
Mokha *et al* 41	Male	20	20.4(±1.3)	23.5	NCAA Division II collegiate athletes	The injury occurred in a practice, session or competition, required attention or the athlete sought medical care and resulted in modified training for at least 24 hours or required protective splinting or taping for continued sport participation.
	Female	64	19.1(±1.2)	22.6		
O’Connor *et al* 35	Male	874	22.4 (±2.7)	–	Marine officer candidates	Physical damage during training and sought medical care one or more times. It included all injury cases. Overuse injuries were long-term repetitive energy exchange, and serious injuries were any type of injury that was severe enough to remove the subject from the training programme.
Warren *et al* 40	Male	89	20.0	23.9–25.9	NCAA Division I collegiate athletes	Non-contact mechanism that was reported to the athletic training room and required intervention
	Female	78				

–, not reported.

*Approximately calculated based on the data provided by the author.

BMI, body mass index; NCAA, National Collegiate Athletic Association.

The definition of injury was explained in all the studies. Two studies were stratified according to the type of injury, and these studies divided and defined the injury as any injury, overuse injury, traumatic injury and serious injury.

### Summary of effect sizes

Table 3 shows the follow-up, the number of subjects in each group according to their FMS score, the injuries that took place in each group, and the main results of each study, such as sensitivity, specificity, area under the ROC curve and the OR. The type of injury collected in the studies was specified.

**Table 3:** Results from the included studies

	Follow-up	Type of injury	Injured (n)	Non-injured (n)	Sensitivity (95% CI)	Specificity (95% CI)	Area under the ROC curve	OR (95% CI)
Bond *et al* 36	1 season	A	≤14=8* ≥15=48*	≤14=9 ≥15=54	0.14 (0.06 to 0.26)	0.85 (0.74 to 0.93)	0.46 (0.35–0.56)	1.00 (0.36 to 2.80)
Bushman *et al* 37	24 weeks	A	≤14=308 ≥15=612	≤14=283 ≥15=1273	0.33	0.82	0.60	**2.26** **(1.87 to** **2.73**)
		B	≤14=256 ≥15=442	≤14=335 ≥15=1443	0.37	0.81	0.61	**2.49** **(2.05 to** **3.03**)
		C	≤14=110 ≥15=278	≤14=481 ≥15=1607	0.28	0.77	0.54	**1.32** **(1.03 to** **1.68**)
Butler *et al* 34	16 weeks	A	≤14=66* ≥15=13*	≤14=11* ≥15=18*	0.83	0.62	–	**8.31** **(3.20 to** **21.63**)
Chorba *et al* 32	1 season	A	≤14=11 ≥15=8	≤14=5 ≥15=14	0.58 (0.34 to 0.80)	0.74 (0.49 to 0.91)	–	3.85 (0.98 to 15.13)
Dossa *et al* 33	1 season	A	≤14=5 ≥15=5	≤14=3 ≥15=7	0.50 (0.19 to 0.81)	0.70 (0.35 to 0.93)	–	2.33 (0.37 to 14.61)
Garrison *et al* 42	1 season	A	–	–	0.67	0.73	–	**5.61** **(2.73 to** **11.51**)
Hotta *et al* 39	24 weeks	A	≤14=11* ≥15=4*	≤14=32 ≥15=37	0.73	0.46	0.65	3.20 (0.90 to 11.00)
Kiesel *et al* 9	18 weeks	A	≤14=7 ≥15=6	≤14=3 ≥15=30	0.54 (0.34 to 0.68)	0.91 (0.83 to 0.96)	–	**11.67** **(2.47 to** **58.52**)
Knapik *et al* (male)^31^	8 weeks	A	≤14=79 ≥15=64	≤14=321 ≥15=306	0.55	0.48	0.53	1.18 (0.82 to 1.69)
Knapik *et al* (female)^31^	8 weeks	A	≤14=41 ≥15=27	≤14=80 ≥15=127	0.60	0.61	0.59	**2.41** **(1.38 to** **4.22**)
Kodesh *et al* 38	12 weeks	A	≤14=41* ≥15=56*	≤14=22 ≥15=39	0.42	0.63	0.51	1.29 (0.67 to 2.51)
Mokha *et al* 41	6 months	A	≤14=10 ≥15=28	≤14=19 ≥15=27	0.26	0.58	–	0.51 (0.20 to 1.29)
O’Connor *et al* 35	6–10 weeks	A	≤14=42* ≥15=228*	≤14=51* ≥15=553*	0.45	0.78	0.58	**2.00** **(1.30 to** **3.10**)
		B	≤14=12* ≥15=78*	≤14=79* ≥15=703*	0.12	0.90	0.52	1.40 (0.71 to 2.60)
		D	≤14=11* ≥15 = 48*	≤14=80* ≥15=733*	0.11	0.93	0.53	**2.00** **(1.00 to** **4.10**)
Warren *et al* 40	16 weeks	A	≤14=40* ≥15=34*	≤14=50* ≥15=43*	0.54	0.46	0.48	1.01 (0.53 to 1.91)

Values in bold, statistical significant results; –, not reported.

*Approximately calculated based on the data provided by the author.

A, any; B, overuse; C, traumatic; D, serious; ROC, receiver operating characteristic.

Six studies with a cut-off of 14/21 had an OR with significant results. These OR values were between 2.00 (95% CI 1.00 to 4.10) and 11.67 (95% CI 2.47 to 58.52).

According to the data on the injuries from the studies investigated, the sensitivity was between 0.26 and 0.83 and the specificity was between 0.46 and 0.91. The area under the ROC curve values was between 0.48 and 0.65.

### Summary of the meta-analysis

A 2×2 contingency table composed of participants is shown in table 4.

**Table 4:** A 2×2 contingency table composed of all subjects of the selected studies for the meta-analysis

	Injured	Non-injured
FMS score ≤14	669	889
FMS score ≥15	1133	2528

FMS, Functional Movement Screen.

**Figure 2:**
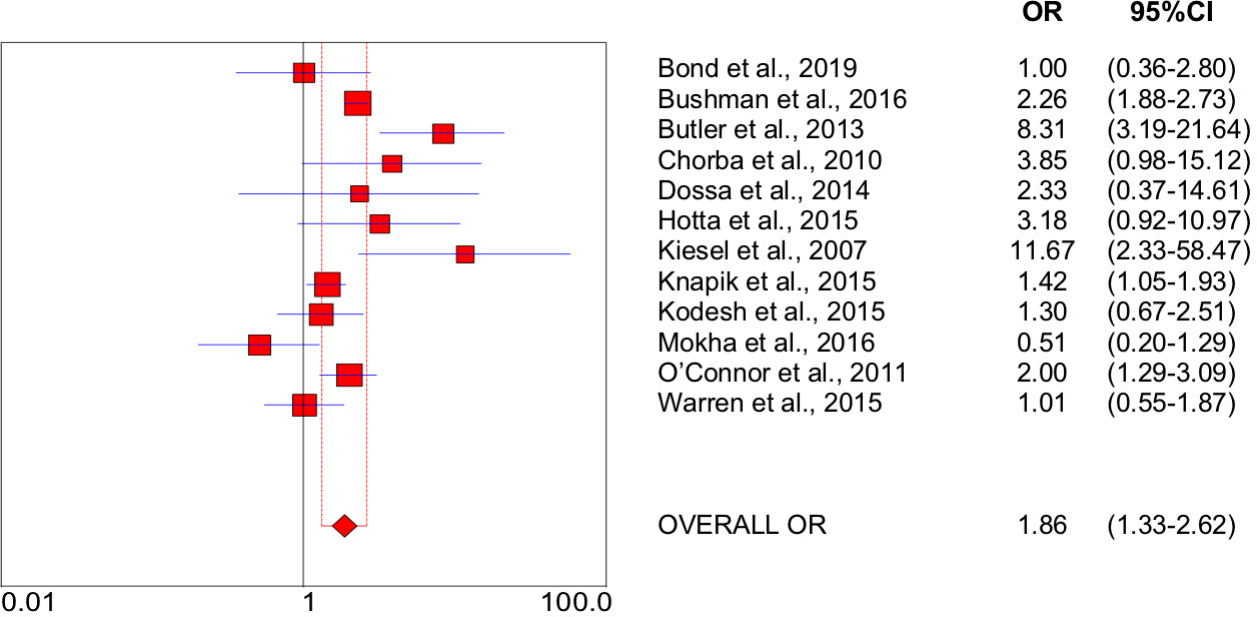
Overall OR of the selected studies for meta-analysis and forest plot. Plot from Meta-DiSc software (Unidad de Bioestadística Clínica del Hospital Ramón Y Cajal, Madrid, Spain).

The meta-analysis and the forest plots are shown in figure 2. The effect sizes of the studies selected for the meta-analysis were tested for heterogeneity, with Q=37.13 and df=11. The other statistical test showed substantial heterogeneity (I^2^=70%). The overall OR of the selected studies was 1.86 (95% CI 1.32 to 2.61).

## DISCUSSION

This systematic review and meta-analysis assessed the literature to determine whether there is an association between an FMS score of less than 14 out of 21 points and subsequent injuries and whether it could serve as a useful tool. The results suggest the association is not clear. It has been observed that the results do not offer strong arguments in favour of the 14/21 cut-off, which is widely used in the literature since the results reported by Kiesel *et al*.^9^ Only half of the studies to date have shown the discriminating use of the FMS. The focus on using a score of 14/21 to avoid the variability in other cut-offs reported by other systematic reviews^23–26^ did not support the association between the FMS and subsequent injuries. However, an analysis of previously published systematic reviews showed that the cut-off was initially set at 14/21, but actually studies with a score of 14 (±3)/21 were analysed. This should suggest the possibility of proposing, instead of a specific cut-off point, a range of scores that allow the screening from a qualitative rather than a quantitative point of view. In this way, functional capacities at different levels could be stratified and the association according to the category analysed.

### Association between FMS score and injuries

Five out of 12 studies, in addition to the female sample from the study of Knapik *et al*,^31^ demonstrated that FMS score was associated with subsequent injuries. This systematic review did not confirm that the 14/21 cut-off was associated with subsequent injuries. According to another systematic review, 6 of 15 results on the association between FMS score and injury risk (OR or risk ratio) showed a significant effect.^23^ Perhaps performing a stratification by levels (integrating different cut-off points) of functional capabilities of the subject could be a qualitative screening method that facilitates the functional analysis (and association with injuries) of the FMS.

One of the possible reasons for these differences may be the definition of the injury in each article. The different criteria used by the different studies make it very difficult to compare across them and helps to understand the poor evidence that was represented in the systematic reviews. For example, we could divide the definitions according to medical criteria^31 32 35 38 40^ or according to the time since the last sports activity.^9 33 34 36 39 41 42^ These criteria are very difficult to compare; in addition, the time since last sports activity ranges from 1 day to 4 weeks according to each article. Also, the fact that they do not take into account previous injuries cannot be ignored. A review in 2014 regarding injury risk and runners concluded that the main indicator of risk was being injured in the previous 12 months.^43^ A previous injury could influence the FMS score because it is possible that subjects will score worse. To improve the comparison, the definition of injury must follow the same standards according to a reference test.

The different samples and follow-up used in each study did not influence the results. If we divide studies into short-term follow-up (0–4 months)^31 34 35 38 40^ and long-term follow-up (4 months–1 season),^9 32 33 35 37 39 41 42^ the results that are in favour of the FMS having an association with the likelihood of injury were distributed in both groups, so it was impossible to define the tendency of a relationship with injuries according to follow-up. The risk of bias in the short-term follow-up studies was low,^23^ and only one study did not obtain results in favour of the FMS. Despite the advantage of short-term follow-up, other methodological factors have been more decisive. With respect to the sample size, there are inconclusive relationships as with the follow-up. The follow-up and the sample size are important factors to take into account in subsequent studies.

Gender differences and the risk of injury have been studied in the literature.^44 45^ According to the results by gender, the FMS score was a significant risk factor in women (OR=2.41, 95% CI 1.38 to 4.22) who participated in the study of Knapik *et al*.^31^ However, for men who participated in the sample of the same study, the FMS score was not a risk factor (OR=1.18, 95% CI 0.82 to 1.69). If these results are compared with Chorba *et al* 32 and Kodesh *et al*,^38^ FMS score was not a significant risk factor for women in both studies (OR=4.58, 95% CI 0.99 to 21.13 and OR=1.29, 95% CI 0.67 to 2.51). A notable difference between the two samples was that the study sample of Knapik *et al* 31 was bigger (n=275) than that of Chorba *et al* 32 (n=38) and Kodesh *et al*.^38^ In short, methodological biases would have to be minimised to better interpret the results^46^ because the comparison between men and women is important to confirm that injury prevention strategies should be specific to each gender.^47^

Finally, the variability in the sensitivity (0.26–0.83) and specificity (0.46–0.91) is huge, and previous studies have rendered the 14/21 score doubtful. Most of the selected studies decided to find a better cut-off, and a sensitivity and specificity analysis of other cut-offs was carried out. Within the studies selected in this systematic review, Mokha *et al* 41 obtained a better sensitivity (0.83) and specificity (0.88) with 16 as the cut-off, and Knapik *et al* 31 with a cut-off score of 12 in the male sample obtained a sensitivity of 0.22 and specificity of 0.87. There are other examples in the literature with other cut-offs with better sensitivity and specificity, such as Letafatkar *et al* 48 which used a cut-off of 17 with good sensitivity (0.64) and specificity (0.78). On the contrary, there are no firm results that establish an acceptable cut-off point with excellent results^49^ in sensitivity and specificity except for the study of Mokha *et al*.^41^ Therefore, a sensitivity and specificity analysis in each study is a good option to find the best cut-off due to the variability in design, as has been shown before.

### Homogeneity of the meta-analysis

The meta-analysis from the systematic review confirmed that there was some heterogeneity in the selected results. The selected studies reflected substantial heterogeneity (I^2^=70%) according to Higgins and Green,^30^ as the value was between 50% and 90%. The differences discussed above could create great variability which could be caused by the level of heterogeneity, so the overall OR (2.03, 95% CI 1.23 to 3.35) is not a valid value due to the poor homogeneity of the selected studies.

If other systematic reviews on the FMS and subsequent injuries are compared with the current study, both come to an agreement that the precision of the FMS for prediction of the risk of injury is low and that its effectiveness could not be verified. Dorrel *et al* 26 performed the first systematic review on the FMS, and a conclusion was obtained after a meta-analysis of the diagnostic reliability, which showed that the FMS had low sensitivity (0.24) and good specificity (85.7). The authors proposed that each study should look for its cut-off according to its sample and definition of the lesion since, as it has been confirmed in this review, using the same cut-off score does not achieve better solid results.^26^ When compared with the results of Moran *et al*,^23^ the current review obtained better results in the meta-analysis (OR=1.47, 95% CI 1.22 to 1.77, I^2^=57%), but they used three studies and a military male sample. Having similar samples and the definition of the lesion very close, by medical decision, confirmed the importance of having minimum heterogeneity in the studies, emphasising that there was no uniformity in the selected articles in the three reviews. The meta-analysis outcomes presented in this review of 12 studies, with a larger number of different samples and with both genders included, increased the heterogeneity. To conclude, there are no findings that support a relationship between FMS score and injury risk.

The lack of consensus is common in this kind of tools because there are other tests that need to be further investigated.^50 51^ Lisman *et al* 52 and O’Connor *et al* 35 used Physical Fitness Tests (PFTs) along with FMS. A PFT score of less than 280 was a risk factor (OR=2.1, 95% CI 1.5 to 2.9) with a male sample. Lisman *et al* 52 specified that a PFT that could significantly predict a risk factor was the 3-mile run in more than 20.5 min (OR=1.72, 95% CI 1.29 to 2.31). In contrast, the PFT had limited and conflicting evidence regarding reliability and validity in common usage.^53^ Future studies should better define the sample, the definition of the injury and the methodology because the FMS is far from being a good tool to identify a high risk of injury to the individual.^54^

### Methodological quality

We noted a wide range in the methodological quality of the studies that comprised our systematic review. There was too much difference between the scores used as a reference in the different studies. Theoretically, the cut-off should be 14, but they actually ranged from 11 to 18. Importantly the five studies that had the poorest method scores were those studies that confirmed the relationship between a low FMS score and injury.^9 31 34 37 42^ According to Bahr,^55^ the majority of studies on injury prediction were inappropriately designed because they did not explain the causative factor with sufficient accuracy. Therefore, the lack of good methodology could influence the information, and the limited methodology could detriment the results of the systematic review and the heterogeneity of the meta-analysis.

### Clinical importance

Although many sports teams use the FMS at the beginning of the season, there is no evidence to confirm its association with injuries. Sport has been the starting point to identify the importance of having good tools to predict injuries; however, other types of assessments must deal with physically demanding tasks.^56^ Most of the selected studies included samples related to sports or workers that required good physical condition. A good initial assessment could help reduce the time lost in a competition or work, and reduce the costs associated with injuries.^57^

Due to its ability to evaluate stability and strength, and because it is easy to administer and perform and is very adaptable to the clinical environment,^13 14^ the FMS could be a good tool for clinicians and physiotherapists. Despite the advantages that the FMS may have in clinical practice, according to the selected studies the association between FMS score and injuries is limited, and due to the heterogeneity of the data there can be no consensus on what should be the reference score to use for the FMS as a predictive tool for injuries. Therefore, it is necessary to focus on the nature of the patient in daily practice and individualise the clinical information collected.

### Limitations

The study has some limitations. The number of studies included in the review was small. The meta-analysis showed that the OR of the selected studies had a heterogeneous distribution. The definition of injury was not very similar in the studies and could be a reason for this heterogeneity. From a methodological point of view, we used the most well-known appraisal tool for observational studies—STROBE. Other tools more focused on diagnosis could be more adjusted to the topic, but since the FMS is not a conventional diagnostic tool we could clearly and concisely score and classify the quality of the articles included using STROBE.

The samples used in the studies were quite similar because subjects were young and were in sports or had a very good physical condition. It would be interesting to see how it affects other people, because the risk of injury increases with age,^58^ and using participants who are not involved in sports in prospective studies.

## CONCLUSION

This systematic review shows that the relationship between the FMS score and injury is unclear. Half of the studies showed that a low FMS score was statistically associated with risk of sports injury. The heterogeneity of the study populations (type of athletes, age and sport exposure) and the definition of injury used in the studies make it difficult to synthesise the evidence and draw definitive conclusions.
